# Intrusion Detection Model for Industrial Internet of Things Based on Improved Autoencoder

**DOI:** 10.1155/2022/1406214

**Published:** 2022-05-27

**Authors:** Wumei Zhang, Yongzhen Zhang

**Affiliations:** Zhejiang Tongji Vocational College of Science and Technology, HangZhou, Zhejiang 311231, China

## Abstract

With the gradual advancement of informatization and industrialization, the safety and controllability of industrial Internet of things (IoT) have attracted more and more attention. Aiming to improve the security of industrial IoT, a detection method using stacked sparse autoencoder network model is proposed. In this method, the basic units of the network model have been simplified and sparse, and some of basic features are combined with obtaining a higher-level abstract expression, so as to solve the problem of unbalanced network traffic data. The cascaded network structure is adopted to stack its sparse autoencoder network model, so as to improve the data ability of the detection model. In addition, the incorporation of Softmax classifier realizes the dynamic adjustment and optimization of the whole network parameters, which further ensures the efficiency of the detection method. The simulation experiment is based on NSL-KDD dataset. The experiment has proved that the proposed method has excellent network attack identification and detection performance. Its accuracy index is about 95.42%, and the detection time is about 3.42 s.

## 1. Introduction

The essence of Internet of things (IoT) is the integrated development of industrial automation and interconnection of all things technology [1–3]. The Industrial Internet of things (IIoT) has realized the unprecedented combination of subsystems such as production, monitoring, and management. Different systems can process all kinds of industrial data more efficiently under the unified management of the control center [4, 5]. Its high complexity and openness increase the network security risk faced by the industrial IoT.

Typical network attacks in industrial control systems are common [6]. In July 2010, the first virus “Stuxnet” targeting the Supervisory Control and Data Acquisition (SCADA) system attacked Iran's nuclear facilities. In 2012, the “Flame” virus paralyzed Iran's oil industry network. Since then, the incidents of hacker attacks on industrial control systems have been reported all over the world, and the frequency and impact have shown a rapid upward trend year by year. Industrial control security has become a complex of “network security, equipment security, control security, application security, and data security” [7]. Therefore, it is particularly urgent to propose an accurate and efficient network intrusion detection method.

Intrusion detection system is widely used in traditional industrial control system and modern industrial IoT, and it has attracted more and more attention [8, 9]. In [10], the authors detect attacks on the industrial IoT based on BiLSTM-RNN and use the UNSWNYB15 dataset to train a multilayer neural network. In [11], the authors designed a network intrusion detection system for the SCADA system based on CNN to protect the IIoT from conventional network risk such as DDoS and specific network attacks against SCADA. In [12], the authors studied the power theft attack in the smart grid and proposed a detection method using the multilayer network. However, it should be pointed out that when facing the current high real-time, high-capacity and complex multidimensional data in industrial IoT, the above methods often need a complex training process, and the accuracy needs to be improved [13].

Deep network can not only obtain the maximum reward from the high-dimensional and massive network data environment but also have the exploration function and automatically mine more valuable information in the network environment [14–16]. Therefore, many scholars have carried out research studies and analyses using deep learning network. In [17], the authors used a context adaptive intrusion detection system, which realizes the accurate detection of network attacks through the mutual assistance of multiple agents. The IIoT detection model in [18] combines feedforward neural network and long-term and short-term memory network. In [19], the authors used an IIoT detection model based on intelligent algorithm and multilayer network, which can achieve better detection efficiency. In [20], the authors proposed a new multiagent confrontation reinforcement learning model for IIoT detection system to realize steady-state support for the network environment. However, it should be noted that the industrial IoT data has unbalanced characteristics. The current deep learning intrusion detection method cannot achieve accurate data feature extraction in the network data with too many feature dimensions, and it is difficult to support efficient and accurate intrusion attack-type mapping. At the same time, due to the deeper network structure, the deep network model also has the problem of time-consuming in intrusion detection.

Aiming at the above problems, based on the improved autoencoder (AE), a detection method for IIoT is proposed. The main innovations are as follows:

1. In this study, the network structure unit of the multilayer network is sparse. By adding sparsity constraints to the hidden layer, some neurons are suppressed, and the problem of industrial network intrusion detection with unbalanced network traffic data is solved, so as to learn more accurate and efficient feature expression.
2. The cascade form is used to combine the sparse autoencoder (SAE) network and construct the stacked sparse autoencoder (SSAE) network model, which can realize the continuous deep feature extraction of industrial IoT network data, so as to support the high accuracy of intrusion detection network.

## 2. Standard Autoencoder Model Learning Algorithm

Industrial control system network dataset presents the characteristics of more normal data, less abnormal data, and uneven data distribution [21]. Algorithms including traditional artificial neural network cannot effectively classify and identify unbalanced data.

AE network is an unsupervised feature detection model, which can learn a feature representation of input data. This model belongs to artificial neural network and is optimized by backpropagation algorithm.

The essence of the algorithm of self-encoder network is an unsupervised training and learning method. In order to make the target value input directly, it introduces the data processing model of backpropagation to maintain the consistency of data.

In addition to being used as the construction module of deep neural network, the AE network can also be used to extract discriminant features with lower dimension than input, so as to solve the dimension disaster.

**Figure 1:**
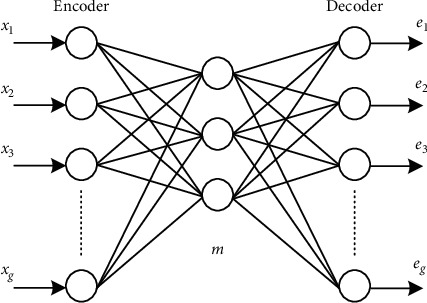
Autocoding network structure.

The standard AE is a multilayer feedforward network, which expects the input and output to be consistent. It can be used to learn identity mapping and extract unsupervised features.  Figure 1 is a network structure of a single-layer autoencoder, in which only one hidden layer is used to encode the input and reconstruct the input at the output through decoding. The part from the input layer to the middle layer is called encoder, and the part from the middle layer to the output layer is called decoder. Autoencoder is an unsupervised feature detection model, which can learn another feature representation of input data. Autoencoder learns to generate a hidden layer representation from the input and reconstructs the output as close to the input as possible from the hidden layer representation.

As can be seen from Figure 1, the AE network model is composed of the input layer, the hidden layer, and the output layer. Specifically, the purpose of the self-encoder is to make the output value of the model equal to or as close to the input value of the model as possible with the help of an identity function. *x*_*t*_=*e*_*t*_.

Encoding refers to the process of mapping input *x* ∈ *R* to implicit representation *h*(*x*) ∈ *R*. The calculation form is

(1) $$\begin{array}{c} h\left(x\right)=\alpha_{h}\left(Wx+b\right), \end{array}$$

where *W* ∈ *R* is the encoding weight matrix, *b* ∈ *R* is the encoding offset vector, *α*_*h*_(*x*) is the vector value function, and in the case of nonlinearity, *α*_*h*_(*x*) is taken as Sigmoid function.

Decoding refers to mapping the implicit representation *α*_*h*_(*x*) to the output layer *e*, so as to reconstruct the input *x*. The calculation form is

(2) $$\begin{array}{c} e=\alpha_{e}\left(Wh\left(x\right)+b^{\prime }\right), \end{array}$$

where *W*′ ∈ *R* presents the decoding matrix, *b*′ ∈ *R* presents the decoding vector, and *α*_*e*_(*x*) is similar to *α*_*h*_(*x*).

## 3. Intrusion Detection Model of IIoT

Excessive feature dimension is the reason for the low efficiency of industrial control safety anomaly detection [22, 23]. Dimension reduction can be achieved by reducing high-dimensional and nonlinear attribute features. Through the sparse expression of features, a small number of basic features are combined to obtain a higher-level abstract expression.

Therefore, based on the standard AE network, this study adds sparsity constraints to the output of the hidden layer so that most neurons are suppressed and constructs a atacked sparse autoencoder (SSAE) network model.

The SSAE network is used to establish the intrusion detection model of the IIoT. On the premise of maintaining the accuracy of detection, the calculation speed and calculation memory are improved, so as to learn better feature expression.

### 3.1. Overall Architecture

**Figure 2:**
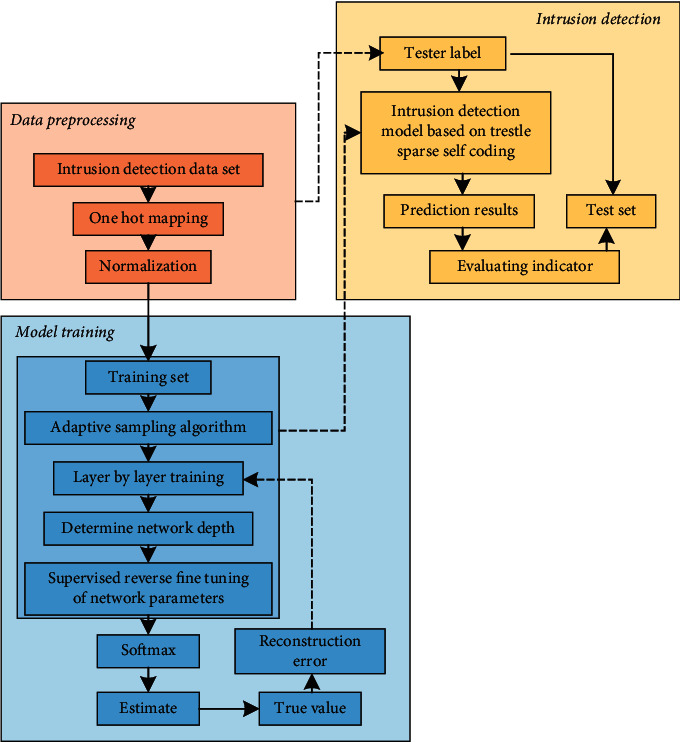
Intrusion detection model for IIoT.

The proposed overall architecture is shown in Figure 2.

From Figure 2, the identification of industrial IoT intrusion by this model mainly includes the following three steps:

1. Data preprocessing: build an industrial IoT environment and capture real-time network data, including source address, target address, connection attributes, and other relevant information [24, 25]. The data are preprocessed and transformed into a format that can be processed by the stacked noise reduction convolutional autoencoder. In this study, data preprocessing is divided into three parts:

① Attribute mapping: convert character data into numerical data
② Data normalization: normalize the data to within 0 to 1 to solve the problem of dimensional inconsistency, which affects the accuracy
③ Regional adaptive oversampling algorithm: generate new samples at the algorithm level for minority samples, handle the imbalance of data distribution properly, and then carry out the next operation to optimize minority data

### 3.2. Stacked Sparse Autoencoder Network

SAE network suppresses most neurons by adding sparsity constraints to the output of the hidden layer, which can learn better feature expression, so as to solve the problem of industrial network intrusion detection with unbalanced network traffic data. The specific way is to add a sparse penalty term, that is, the function of the average output activation value of neurons.

The goal of SAE is to make the output fit the input features, which is similar to AE, but SAE imposes sparsity restrictions on the middle layer in order to avoid simple mapping output to input.

The simple understanding of sparsity restriction is that when the output of neuron in each layer is 0, it indicates that the state of neuron is inhibited; when the output of neuron is 1, it indicates that the state of neuron is active, and the sparsity restriction makes the state of neuron inhibited most of the time.

The mean activation degree of hidden layer neuron *i* is defined as follows:

(3) $$\begin{array}{c} {\hat{\tau }}_{i}=\frac{1}{n}\sum_{p=1}^{n}\left[c_{i}^{\left(2\right)}\left(v^{\left(i\right)}\right)\right], \end{array}$$

where *n* indicates the total number of data sample sets and *c*_*i*_^(2)^ is the activation parameter of the middle layer neuron *i* when *v* is used as input. To get the sparse representation of the middle layer neuron, it should make the activation mean ${\hat{\tau }}_{i}$ of the middle layer neuron *i* as 0 as possible. If making ${\hat{\tau }}_{i}=\tau$ as a sparsity parameter, *τ* should be a decimal close to zero. By introducing a penalty factor into the solution of the objective, those scenarios that ${\hat{\tau }}_{i}$ and *τ* are significantly different are punished, so as to realize such sparsity limitation and continuously optimize the value of the objective function. There are many ways to construct penalty factors. Here, the Kullback–Leible (KL) is used to regularize the network so that the average activation degree ${\hat{\tau }}_{i}$ is equal to *τ* as much as possible:

(4) $$\begin{array}{c} KL\left(\tau \|{\hat{\tau }}_{i}\right)=\tau \;\log\frac{\tau }{{\hat{\tau }}_{i}}+\left(1-\tau \right)\log\frac{1-\tau }{1-{\hat{\tau }}_{i}}. \end{array}$$

The penalty factor formula is as follows:

(5) $$\begin{array}{c} \sum_{i=1}^{z^{2}}\tau \;\log\frac{\tau }{{\hat{\tau }}_{i}}+\left(1-\tau \right)\log\frac{1-\tau }{1-{\hat{\tau }}_{i}}, \end{array}$$

where *z*^2^ is the sum of neuron. The above penalty factor can also be expressed as $\sum_{i=1}^{z^{2}}KL\left(\tau \|{\hat{\tau }}_{i}\right)$.

It can be seen that the loss function of the detection network is

(6) $$\begin{array}{c} \theta_{sparse}\left(W,b\right)=\theta_{E}\left(W,b\right)+\mu \sum_{i=1}^{z^{2}}KL\left(\tau \|{\hat{\tau }}_{i}\right). \end{array}$$

Usually, in order to avoid the overfitting problem, the *L*_2_ weight penalty is introduced to the objective function; then,

(7) $$\begin{array}{c} \theta_{SAE}\left(W,b\right)=\theta_{\text{sparse}}\left(W,b\right)+\frac{\gamma }{2}\sum_{i=1}^{s_{q}}\sum_{p=1}^{s_{q}+1}\sum_{q=1}^{s_{q}-1}{\left(u_{pi}^{\left(q\right)}\right)}^{2}, \end{array}$$

where *γ* represents the regularization parameter, *q* represents the current layer, and *s*_*q*_ and *s*_*q*_+1 are the sum of neurons.

The formula of descent optimization is as follows:

(8) $$\begin{array}{c} W_{pi}^{\left(q\right)}=u_{pi}^{\left(q\right)}-\psi \frac{\partial }{\partial u_{p}^{\left(q\right)}}\theta_{SAE}\left(W,b\right), \end{array}$$

(9) $$\begin{array}{c} b_{pi}^{\left(q\right)}=b_{pi}^{\left(q\right)}-\psi \frac{\partial }{\partial b_{p}^{\left(q\right)}}\theta_{SAE}\left(W,b\right), \end{array}$$

where *ψ* is the learning rate. The optimal *W* and *b* can be obtained by back propagation using the SGD optimization method.

**Figure 3:**
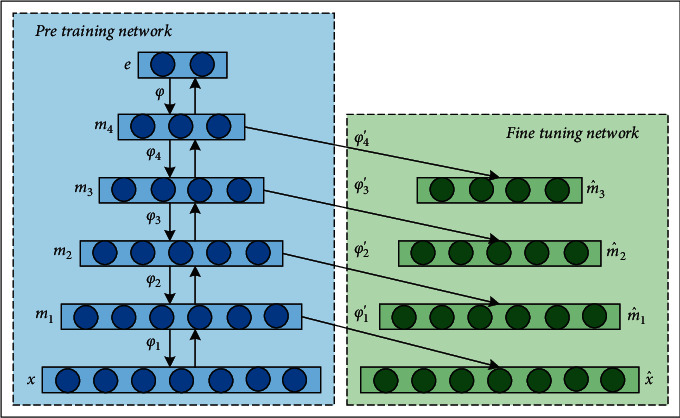
Stacked sparse autoencoder network training process.

The training process of SSAE network is shown in Figure 3.

The first SAE contains layers *x*, *m*_1_, and $\hat{x}$, uses formula (6) to learn the representation of features in an unsupervised manner, and then obtains *U*_1_ and *c*_1_ through formulae (7) and (8) training. The second SAE contains layers *m*_1_, *m*_2_, and ${\hat{m}}_{1}$. The training method of the second SAE is similar to that of the first SAE, and *U*_2_ and *c*_2_ are obtained through training. By repeating the above training steps, all the parameters in the stacked sparse autoencoder network can be obtained.

The way of weight assignment of neural network through pretraining is better than that of random weight assignment of neural network, and it is conducive to convergence. In the training process, the number of neurons decreases gradually, and finally, the deep sparse feature is obtained.

### 3.3. Detection Model Training

Softmax classifier is added in the last layer of SSAE network, and the trained parameters are used as the initial optimization parameters of the model, and then, the parameters of the whole network are fine tuned. This layer-by-layer greedy process is proved to produce a better local extremum than random initialization weights and achieves better generalization performance in some tasks.

The proposed detection model used the SSAE network model is as follows (Algorithm 1).

## 4. Experiment and Result Discussion

### 4.1. Simulation Environment

Tensorflow and OpcnAlGym are the mainstream machine learning training platforms and environments. We choose them as the software environment for simulation experiments. Meanwhile, the experimental hardware environment is CPU model: AMD Ryzen 7, CPU: NVIDIA GeForce RTX2080Ti, and RAM: 32 GB.

### 4.2. Data Preprocessing

At present, the public datasets of industrial IoT intrusion mainly include KDDCup99, NSL-KDD, GasPipeline Datasets, WaterDatasets, and UNSW-NB15. These datasets have the problems of redundancy and repetition of data and attributes. This study selects NSL-KDD dataset as the experimental benchmark data.

NSL-KDD dataset solves the problem of redundant data in KDDCup99 dataset. Its original training set KDDTrain contains 125973 data and the original test set KDDTest contains 22544 data. In this study, KDDTrain+20% of 25192 data are selected as experimental data.

#### 4.2.1. Character-Type Mapping Numeric Type

**Figure 4:**
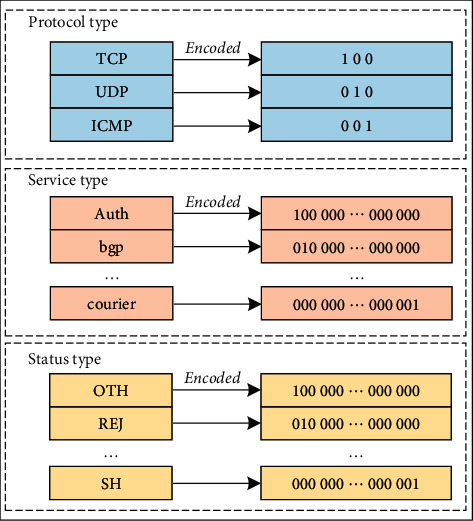
One-hot coding digitization.

“O, tcp, ftp_data, SF, 491, 0, 0, 0, 0, 0, 0, 0, 0, 0, 0, 0, 0, 0, 0, 0, 0, 0, 2, 2, 0, 0, 0, 0, 1, 0, 0, 150, 25, 0.17, 0.03, 0.17, 0, 0, 0, O.OS, O, Normal” is a piece of data in the dataset. According to the analysis, the values in dimension 2, 3, and 4 of the data are character types and need to be converted into numerical types. For example, there are 3 types in dimension 2 (TCP, UDP, ICMP), 70 types in dimension 3 (“auth,” “bgp,” “courier,” etc.), and 11 types in dimension 4 (“0TH,” “REJ,” “RSTO,” etc.), which are processed according to the one-hot coding in Figure 4 and finally convert the 32 dimension into 256 dimension attributes.

#### 4.2.2. Numerical Normalization

Because data order of magnitude and corresponding value range of different feature attributes are obviously different, in order to facilitate the analysis of experimental results, the Min-Max standardization method is used to uniformly map the numerical data to the [0, 1] interval so that the data is in the same order of magnitude:

(10) $$\begin{array}{c} x_{\text{normal}}=\frac{x-x_{\min}}{x_{\max}-x_{\min}}, \end{array}$$

where *x* is the original eigenvalue of data, *x*_min_ and *x*_max_ represents the minimum and maximum values in the data respectively, and *x*_normal_ represents the new feature value after normalization of each data.

#### 4.2.3. Low-Frequency Sample Processing

Although current industrial IoT attacks show a rapid growth trend, the individual attack categories still belong to the low-frequency category compared with the normal data flow, which makes it difficult to capture their feature records. Moreover, most AI models have obvious classification bias because they aim at the overall classification accuracy of the largest sample. Therefore, this study improves the sampling algorithm and introduces the Regional Adaptive Synthetic Oversampling algorithm (RASmote) to incrementally process low-frequency samples. The algorithm formula is as follows:

(11) $$\begin{array}{c} \eta =\left|X_{n}-X_{l}\right|\\ =\sqrt{\sum_{k=1}{\left(X_{nk}-X_{lk}\right)}^{2}}. \end{array}$$

Euclidean distance is used to calculate the distance of low-frequency samples in the nearest neighbor radius. *n* is the nearest neighbor radius, *X*_*n*_ is the nearest neighbor sample set, *X*_*l*_ is the low-frequency sample, and *X*′ is the new sample set:

(12) $$\begin{array}{c} \left\{\begin{array}{cc} X^{\prime }=0, & 0\leq \eta \leq \frac{n}{2},\\ X^{\prime }=X+\mu \left(0,1\right)\left(\left(\frac{1}{n-\eta }\sum_{i=1}X_{i}\right)-X\right), & \frac{n}{2}<\eta <n,\\ X^{\prime }=X, & \eta =n, \end{array}\right. \end{array}$$

where (1/*n* − *η*∑_*i*=1_*X*_*i*_) is a low-frequency sample.

### 4.3. Evaluation Index

The performance of the SSAE intrusion detection model can be evaluated from two aspects: model comparison and classification detection. The model comparison is mainly compared with traditional intrusion detection technology. The main indexes of system detection include accuracy Acc, precision Pre, recall Re, and F1-score *F*_1_. It should be noted that, for these four indexes, the higher the value, the better the detection performance:

(13) Acc=TP+TNTP+FP+TN+FN,Pre=TPTP+FP,Re=TPTP+FN,F1=2×Pre×RePre+Re,

where *T*_*N*_ is true negative rate, *F*_*P*_ is false positive rate, *F*_*N*_ is false negative rate, and *T*_*P*_ is true positive rate.

### 4.4. Experimental Analysis

KDDTrain+20% data are used as the experimental data, 70% as the training set, and 30% as the test set. The data distribution is shown in Table 1.

Firstly, based on the experimental dataset, the detection, analysis, and research of industrial IoT under different network attacks are carried out for the proposed model. The identification results of attacks are displayed in the under table.

From Table 2, we can see that the proposed model can better complete the task of network defense, and the detection accuracy of Dos and Probe attacks is more than 95%. For R2L and U2R attacks, because of the small volume of training data, the identification accuracy is lower than that of the first two attacks, but it is still more than 85%.

**Figure 5:**
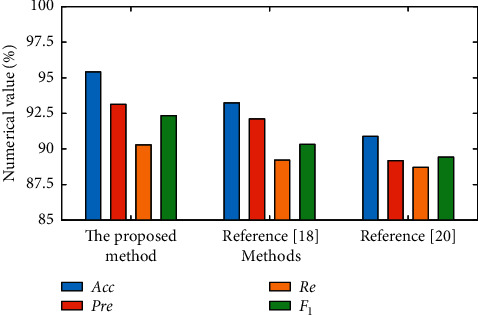
Intrusion detection analysis under different methods.

In order to further verify the performance of the proposed model, the authors [18, 20] are used as comparison methods to detect KDDTrain+20% datasets, respectively.  Figure 5 shows the attack identification results under different intrusion detection methods.

From Figure 5, we can see that the proposed method is better than other comparison methods in terms of network performance. The evaluation indexes of the proposed method are as follows: the accuracy Acc is 95.42%, the precision Pre is 93.14%, the recall Re is 90.29%, and the F1-value *F*_1_ is 92.35%. The accuracy of intrusion detection in [18, 20] is less than 95%, which is less than the detection performance of the proposed method.

The reason is that the proposed model simplifies the network and enhances the autonomous ability and can realize better feature extraction and expression of the network. Meanwhile, with the introduction of Softmax classifier, the detection network parameters can be dynamically adjusted to support accurate network attack identification and analysis. In [18, 20], LSTM network as the benchmark model is taken for modeling and analysis, without considering the imbalance of data, which is not enough to achieve more accurate and efficient intrusion identification analysis.

**Figure 6:**
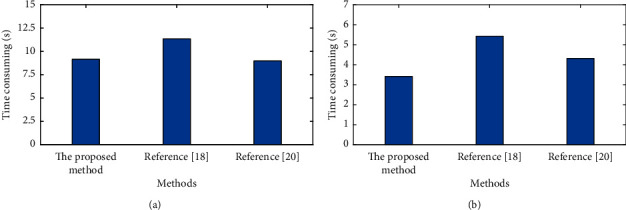
Comparison of intrusion detection time under different methods. (a) Training set time consuming. (b) Test set time consuming.

At the same time, the attack detection efficiency is also compared and evaluated.  Figure 6 shows the analysis of detection time under different methods.

As shown in Figure 6, due to the simplification of the network unit, the unit structure of the proposed method needs more autonomous learning time to realize the accurate extraction of data features. Therefore, the training time is 9.16 s, which is 0.17 s more than that in [20]. Moreover, the time-consuming of the proposed method for network intrusion detection is only 3.42 s and that of [18] is 5.43 s and that of reference [20] is 4.32 s.

To sum up, while ensuring the accuracy of detection, the proposed method can improve the efficiency of intrusion identification and analysis and reflect its overall efficient performance.

## 5. Conclusion

This study proposes an intrusion detection method based on stacked sparse autoencoder network. This method constructs an intrusion network model based on autoencoder network, which can effectively improve the feature extraction of industrial Internet data. The autoencoder network is simplified and cascaded, and a small number of basic network units are used to obtain more efficient feature expression. In addition, the introduction of Softmax classifier ensures that the parameters of the detection network can be fine-tuned and optimized, which can further improve the processing and computing efficiency of the network while improving the accuracy of industrial IoT attack recognition. The experimental analysis based on NSL-KDD dataset shows that the proposed method can realize accurate and fast intrusion attack identification and can meet the safe and controllable operation requirements of industrial IoT.

Although this method improves the solution of IIoT intrusion detection, the essence of the proposed model is a centralized processing and computing model. Aiming to support the detection research in the actual complex network environment, the next step will be to study the intrusion detection method of distributed architecture mode.

## Figures and Tables

**Algorithm 1:**
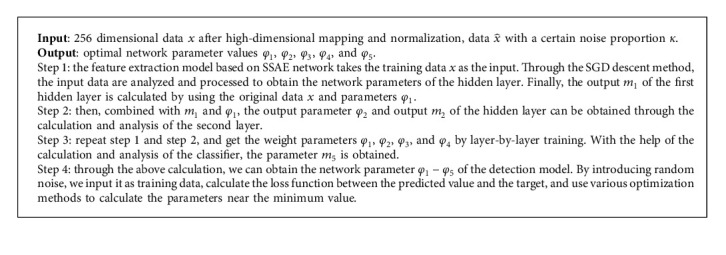
Training algorithm of intrusion detection model based on SSAE network model.

**Table 1:** Distribution of dataset.

Data type	Training set	Test set
Normal	9415	4034
Dos	6500	2734
Probe	1603	786
R2L	145	64
U2R	8	3

**Table 2:** Identification result of different types of network attacks.

Data type	Accuracy (%)
Dos	97.34
Probe	96.81
R2L	91.32
U2R	88.23

## Data Availability

The data used to support the findings of this study are included within the article.
